# Association between protein diet score and colorectal adenomas risk: a prospective study

**DOI:** 10.3389/fimmu.2025.1529011

**Published:** 2025-06-26

**Authors:** Yangpiaoyi Shi, Zhiquan Xu, Wanhao Tan, Hang Liu, Qi Wei, Yaxu Wang, Ling Xiang, Linglong Peng, Haitao Gu

**Affiliations:** ^1^ Department of Gastrointestinal Surgery, The Second Affiliated Hospital of Chongqing Medical University, Chongqing, China; ^2^ Department of Hepatobiliary Surgery, The First Affiliated Hospital of Chongqing Medical University, Chongqing, China; ^3^ Department of Clinical Nutrition, The Second Affiliated Hospital of Chongqing Medical University, Chongqing, China

**Keywords:** protein, protein diet score, colorectal adenoma, epidemiology, prospective study

## Abstract

**Objective:**

Given the significantly increased risk of colorectal adenoma in middle-aged and elderly populations, identifying modifiable risk factors remains a priority. While dietary protein is an essential nutrient in human metabolism, its relationship with colorectal adenoma remains controversial. With advances in nutritional science, contemporary dietary guidelines advocate increasing plant-based protein intake to achieve a more balanced protein consumption pattern. To provide new insights, we sought to investigate the association between colorectal adenoma risk and the Protein Diet Score, which comprehensively evaluates both protein intake and sources.

**Methods:**

This analysis was based on data from the Prostate, Lung, Colorectal, and Ovarian (PLCO) Cancer Screening Trial. The Cox proportional hazards regression model was utilized to compute the hazard ratios (HRs) and 95% confidence intervals (CIs). Restricted cubic spline was employed to illustrate the variation in colorectal adenoma risk across the entire spectrum of the Protein Diet Score. Additionally, subgroup analyses were conducted to ascertain possible effect modifiers, and several sensitivity analyses were performed to evaluate the robustness of the findings.

**Results:**

During the mean follow-up period of 11.0 years, 992 newly diagnosed colorectal adenomas were identified. In the fully adjustment for potential confounders, the inverse association between Protein Diet Score and colorectal adenoma risk remained statistically significant with an HR of 0.81 (95% CI: 0.67-0.99; P_trend_ =0.005) comparing the highest versus lowest quartile. Restricted cubic spline analysis revealed a linear inverse relationship between Protein Diet Score and colorectal adenoma risk (P for nonlinearity =0.317). In the subgroup analyses, we observed a more pronounced inverse association between Protein Diet Score and colorectal adenoma among participants with a history of hypertension (HR _Quartile 4 vs. Quartile 1_: 0.60; 95% CI: 0.43-0.85; P_interaction_ =0.017). Finally, a series of sensitivity analyses strengthened the robustness of our findings.

**Conclusion:**

Our findings indicate that higher Protein Diet Score is associated with reduced colorectal adenoma incidence among middle-aged and elderly Americans, with similar findings observed for the PAR. These results provide important evidence for optimizing protein intake and source composition to promote intestinal health.

## Introduction

The majority of colorectal cancer (CRC) cases develop through the adenoma-carcinoma sequence, with colorectal adenoma serving as the primary precursor lesion (1). Meanwhile, age-related physiological decline and immune dysfunction increase colorectal adenoma risk, particularly affecting middle-aged and elderly individuals. Therefore, identifying modifiable factors influencing colorectal adenoma development is increasingly important.

Previous investigations have established potential associations between various essential nutrients (including carbohydrates, lipids, vitamins, and minerals) and colorectal adenoma risk (2–6). Although protein represents an essential nutrient, its role in colorectal adenoma development remains controversial (7). Emerging evidence demonstrates its dual role: while moderate protein intake appears beneficial in maintaining intestinal homeostasis through regulation of inflammatory responses and immune function (8–10). Excessive protein intake may promote colorectal tumor risk by increasing serum insulin-like growth factor I (IGF-I) levels, which induces anti-apoptotic effects and excessive cell proliferation in colorectal cells (11, 12).

With advances in nutritional science, contemporary dietary guidelines advocate increasing plant-based protein intake to achieve a more balanced protein consumption pattern (13). This recommendation may stem from the potentially differential effects of proteins from various sources on intestinal health (14). Recently, a novel protein assessment tool - the Protein Diet Score - has been developed to comprehensively evaluate both protein intake and sources (15). Therefore, we aim to investigate the association between Protein Diet Score and colorectal adenoma incidence among middle-aged and elderly populations utilizing a large-scale prospective database from the United States.

## Methods

### Study design

This prospective cohort data was derived from the Prostate, Lung, Colorectal and Ovarian (PLCO) Cancer Screening Trial, a randomized controlled trial designed to evaluate the efficacy of screening tests in reducing mortality from colorectal, lung, prostate, and ovarian cancers. Between November 1993 and July 2001, approximately 155,000 participants aged 55–74 years were enrolled across ten screening centers throughout the United States. Participants were randomized into intervention and control arms, with control arm participants receiving usual medical care. In contrast, intervention arm participants underwent screening examinations for prostate, lung, colorectal, and ovarian cancers according to the study protocol. The PLCO protocol was approved by the Institutional Review Boards of all participating centers and the National Cancer Institute (NCI). Written informed consent was obtained from all participants. For this analysis, we utilized the publicly available dataset approved by the NCI (Project ID: PLCO-1724).

### Population for analysis

**Figure 1:**
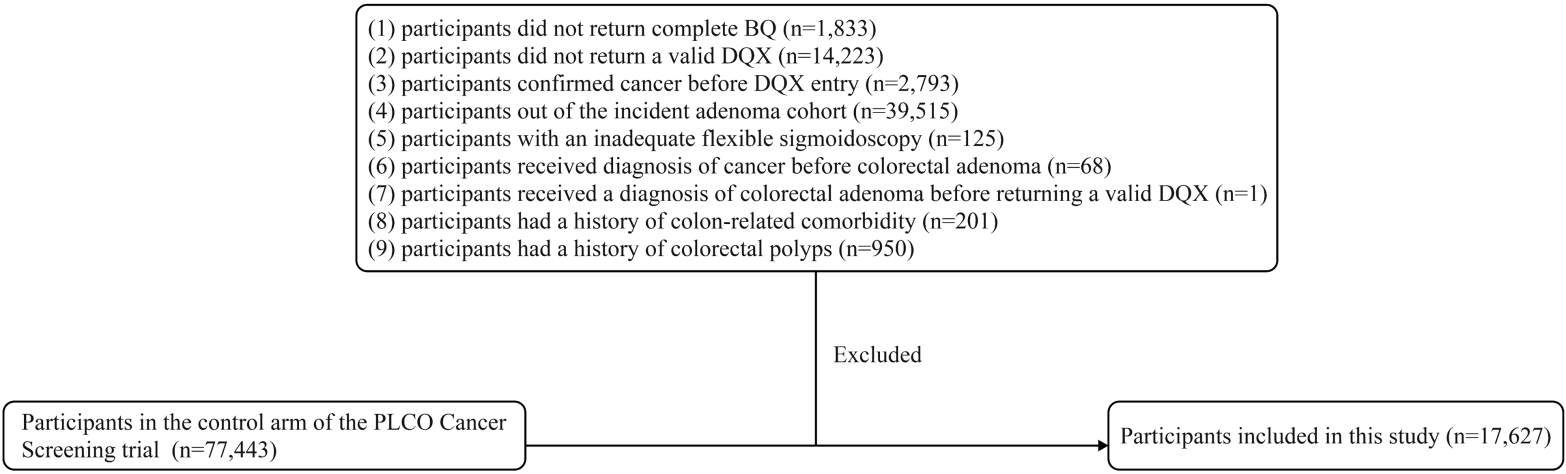
The flow chart of identifying subjects included in our study. PLCO, prostate, lung, colorectal, and ovarian; BQ, baseline questionnaire; DQX, dietary questionnaire.

In the present study, we focused on the incidence of conventional colorectal adenoma as our primary outcome. Therefore, we included participants from the control arm who underwent colorectal cancer screening examinations. We further excluded participants based on the following criteria: (1) did not return complete Baseline Questionnaire (BQ) (n=1,833); (2) did not return a valid Dietary Questionnaire (DQX). Invalid DQX was defined as: 1) completion date missing or postdated to death; 2) ≥8 missing frequency responses; 3) extreme calorie intake by gender (n=14,223); (3) confirmed cancer before DQX entry (n=2,793); (4) out of the incident adenoma cohort (the identification: a negative screen at baseline and either a negative screen at T3/T5 or a positive screen at T3/T5 with a left-sided adenoma found on follow-up to the screen) (n=39,515); (5) with an inadequate flexible sigmoidoscopy (insertion ≥50cm with ≥90% of mucosa visualized) (n=125); (6) received diagnosis of cancer before colorectal adenoma (n=68); (7) received a diagnosis of colorectal adenoma before returning a valid DQX (n=1); (8) had a history of colon-related comorbidity (such as Gardner’s syndrome, ulcerative colitis, Crohn’s disease or familial polyposis) (n=201); (9) had a history of colorectal polyps (n=950). Finally, a total of 17,627 participants were included in the final analytical cohort (**Figure 1**).

### Data collection and covariates assessment

Baseline participant data in the PLCO trial were collected through the BQ, which captured demographic characteristics and medical history. For our analysis, we utilized data on sex, race, education level, occupation, BMI, pack-years smoked, smoking status, history of diverticulitis or diverticulosis, aspirin using regularly, family history of colorectal cancer, history of hypertension, history of diabetes, and history of colonoscopy in past 3 years. Body Mass Index (BMI) was calculated as weight in kilograms divided by height in meters squared (kg/m²). Pack-years smoked were defined as the number of packs smoked per day multiplied by years of smoking.

Additionally, the DQX was offered to intervention arm participants. The DQX was a food frequency questionnaire (FFQ), requiring participants to recall their average consumption frequency of various food items over the previous year. Raw questionnaire responses were processed into analysis-ready variables in terms of gram intake, pyramid servings, food frequencies per day, Total energy and nutrient intake were calculated by multiplying the nutrient content of standard portions of each food item by the reported consumption frequency and summing across all food items. These nutrient amounts came from databases based on national dietary data (USDA’s 1994–96 Continuing Survey of Food Intakes by Individuals [CSFII], available from the USDA Food Surveys Research Group, or the Nutrition Data Systems for Research (NDS-R) from the University of Minnesota, which has nutrient values not available from the USDA Survey Nutrient Database). Furthermore, information on age, alcohol consumption history, dietary patterns, and energy intake was obtained through the DQX.

### Assessment of conventional colorectal adenoma

The incident adenoma cohort was defined as participants who had a negative baseline colonoscopy screening and underwent at least one additional screening colonoscopy at either T3 or T5. All adenomas detected during screening colonoscopies were biopsied and histologically confirmed. According to the U.S. colonoscopy guidelines, conventional adenomas were categorized as advanced adenomas if they met any of the following criteria: (1) any adenoma ≥1cm, with high-grade dysplasia, or with tubulovillous or villous histology should be considered as advanced adenoma; (2) Non-advanced adenomas were defined as those with diameter <1 cm and without advanced histological features.

### Calculation of protein diet score

The Protein Diet Score comprises the percentage of energy from protein intake and the ratio of plant-to-animal protein (15). Participants were stratified into 11 levels based on their protein intake as a percentage of total energy (E%), with scores ranging from 0 to 10 points assigned to participants from the lowest to highest levels. The plant-to-animal protein ratio (PAR) was scored using the same methodology. The final Protein Diet Score was calculated by summing the scores from these two components, with a theoretical range spanning from 0 to 20. Higher scores indicate both greater E% and higher PAR, while lower scores reflect lower E% and PAR.

### Statistical analysis

In the raw data, we identified varying degrees of missingness among the included covariates. The highest proportion of missing data (3.35%) was observed for history of colonoscopy in past 3 years, while missing rates for all other variables were below 1% (**Supplementary Table 1**). Missing values were imputed using mode imputation for categorical variables and median imputation for continuous variables.

**Figure 2:**
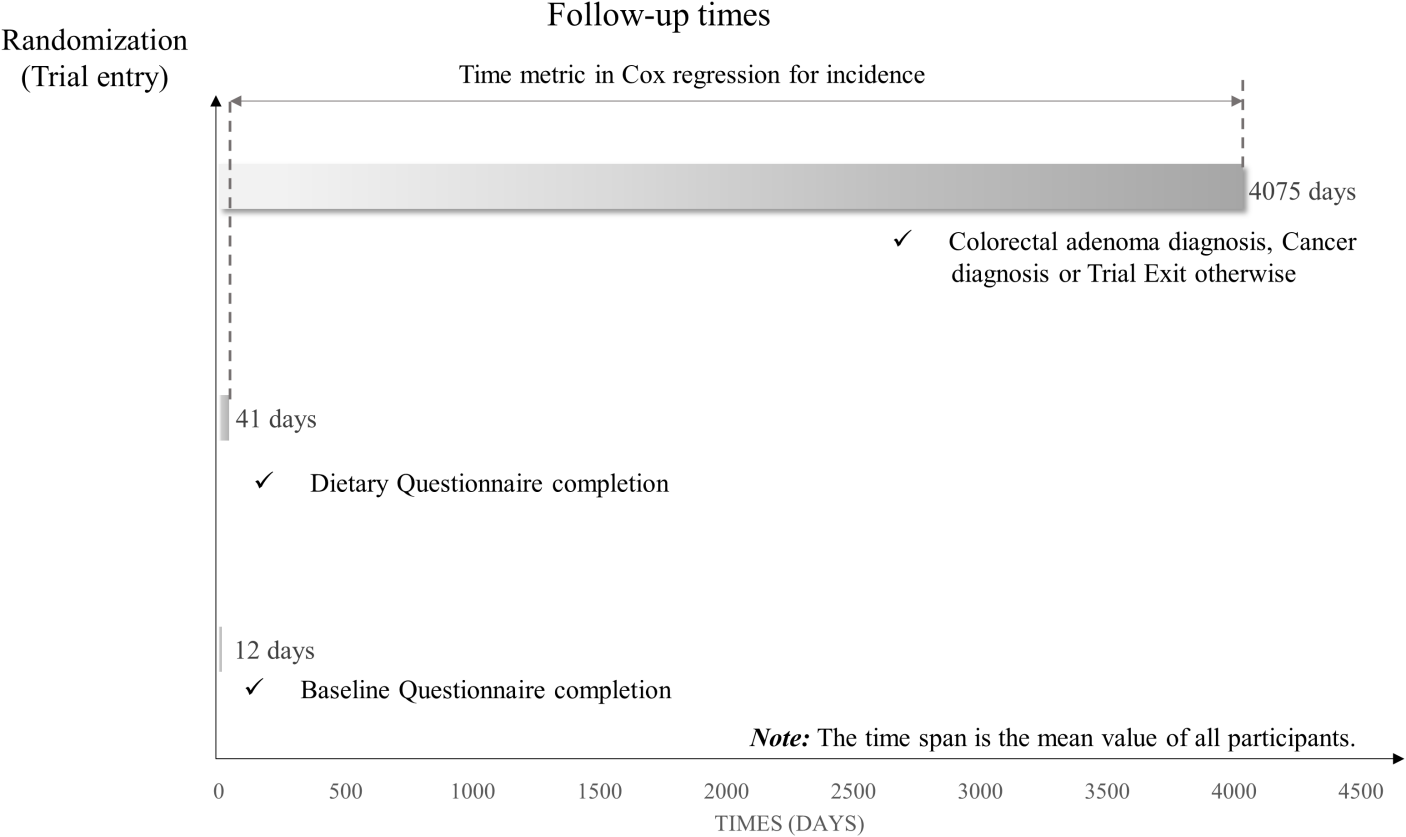
The timeline and follow-up scheme of our study.

To investigate the association between Protein Diet Score and conventional colorectal adenoma, we employed Cox proportional hazards regression models to calculate hazard ratios (HRs) and 95% confidence intervals (95% CIs) with follow-up time as the time variable. Follow-up time was defined as the interval from DQX completion until the first occurrence of any of the following events: diagnosis of conventional colorectal adenoma, cancer diagnosis, death, loss to follow-up, or December 31, 2009 (end of follow-up) (**Figure 2**). In the Cox regression analysis, participants were categorized by quartiles of Protein Diet Score, with the lowest quartile serving as the reference group. To derive P-values for trend, the median Protein Diet Score within each quartile was analyzed as a continuous variable in the Cox models. To account for potential confounding factors, we constructed two multivariate models. Model 1 adjusted for basic demographic characteristics including age, sex, race, education level, occupation and marital status. Model 2 further adjusted for established risk factors: BMI, smoking status, pack-years smoked, drinking status, aspirin using regularly, family history of colorectal cancer, history of diabetes, hypertension, diverticulitis or diverticulosis, and history of colonoscopy in past 3 years. Using the same methodology, we separately evaluated the associations between colorectal adenoma risk and both the E% and the PAR. Potential non-linear associations between Protein Diet Score and conventional colorectal adenoma were assessed using restricted cubic spline models with knots placed at the 10th, 50th, and 90th percentiles.

Likelihood ratio tests were used to examine the multiplicative interaction between Protein Diet Score and pre-specified risk factors in relation to colorectal adenoma risk. Pre-specified risk factors included age, sex, smoking status, family history of colorectal cancer, BMI, aspirin using regularly, and history of hypertension. To assess the robustness of our findings, we conducted several sensitivity analyses: (1) Given the known association between diverticulitis and colorectal adenoma (16), we excluded participants with a history of diverticulitis. (2) To reduce confounding from metabolic abnormalities, we excluded participants with a history of diabetes. (3) To avoid bias from hereditary risk factors, we excluded participants with a family history of colorectal cancer. (4) To minimize confounding effects from other essential nutrients, we further adjusted Model 2 for dietary intake of carbohydrates and fats. (5) Additionally adjusted for foods containing protein nutrients in Model 2 to minimize the impact of other components in food. (6) To minimize reverse causation, we excluded participants who developed outcomes within the first two and four years of follow-up.

All statistical analyses were performed using the R 4.4.1 software and a two-tailed P < 0.05 indicated the significance level.

## Result

### Baseline characteristics

The final study population comprised 17,627 participants with a mean (± SD) baseline age of 62.2 ± 5.2 years. During a median follow-up of 11.04 years, we documented 992 incident cases of colorectal adenoma. **Table 1** presents the baseline characteristics of participants stratified by quartiles of Protein Diet Score. Participants with higher Protein Diet Scores tended to be older, more educated, less likely to be married, had fewer smoking pack-years, and exhibited lower BMI and total energy intake. The proportion of non-white participants, non-drinkers, those without family history of colorectal cancer, and those with history of diabetes increased across ascending quartiles of Protein Diet Score.

**Table 1:** Baseline characteristics of study population according to quartiles of Protein Diet Score.

		Quartiles of overall Protein Diet Score			
Characteristics	Overall	Quartile 1	Quartile 2	Quartile 3	Quartile 4
Protein diet score	10.0 ± 3.1	6.3 ± 1.7	9.6 ± 0.5	11.4 ± 0.5	14.4 ± 1.5
Total protein (E%)	5.0 ± 3.2	3.0 ± 2.3	5.0 ± 3.5	5.8 ± 2.9	7.0 ± 2.0
Plant to animal protein ratio(PAR)	5.0 ± 3.2	3.3 ± 2.3	4.6 ± 3.5	5.7 ± 3.0	7.4 ± 2.0
Age	62.2 ± 5.2	61.9 ± 5.1	62.1 ± 5.2	62.4 ± 5.2	62.7 ± 5.2
Sex					
Male	9868 (56.0%)	3367 (66.3%)	2885 (55.9%)	2003 (50.5%)	1613 (47.2%)
Female	7759 (44.0%)	1713 (33.7%)	2278 (44.1%)	1961 (49.5%)	1807 (52.8%)
Marital status					
Married	14357 (81.4%)	4180 (82.3%)	4246 (82.2%)	3216 (81.1%)	2715 (79.4%)
Unmarried	3270 (18.6%)	900 (17.7%)	917 (17.8%)	748 (18.9%)	705 (20.6%)
Race					
White	15955 (90.5%)	4824 (95.0%)	4751 (92.0%)	3569 (90.0%)	2811 (82.2%)
Non-white	1672 (9.5%)	256 (5.0%)	412 (8.0%)	395 (10.0%)	609 (17.8%)
Education level					
College below	10696 (60.7%)	3324 (65.4%)	3190 (61.8%)	2306 (58.2%)	1876 (54.9%)
College graduate	3263 (18.5%)	887 (17.5%)	946 (18.3%)	755 (19.0%)	675 (19.7%)
Postgraduate	3668 (20.8%)	869 (17.1%)	1027 (19.9%)	903 (22.8%)	869 (25.4%)
Occupation					
Not working	1910 (10.8%)	422 (8.3%)	558 (10.8%)	520 (13.1%)	410 (12.0%)
Working	7539 (42.8%)	2321 (45.7%)	2241 (43.4%)	1634 (41.2%)	1343 (39.3%)
Retired	7483 (42.5%)	2146 (42.2%)	2155 (41.7%)	1648 (41.6%)	1534 (44.9%)
Other	638 (3.6%)	177 (3.5%)	192 (3.7%)	144 (3.6%)	125 (3.7%)
Unknown	57 (0.3%)	14 (0.3%)	17 (0.3%)	18 (0.5%)	8 (0.2%)
Body mass index at baseline (kg/m^2^)	27.1 ± 4.6	27.4 ± 4.3	27.3 ± 4.7	26.9 ± 4.7	26.5 ± 4.5
Smoking status					
No	9379 (53.2%)	2446 (48.1%)	2744 (53.1%)	2207 (55.7%)	1982 (58.0%)
Current	970 (5.5%)	400 (7.9%)	271 (5.2%)	196 (4.9%)	103 (3.0%)
Former	7278 (41.3%)	2234 (44.0%)	2148 (41.6%)	1561 (39.4%)	1335 (39.0%)
Pack-years smoked	13.9 ± 23.5	17.1 ± 26.4	13.6 ± 22.8	12.6 ± 22.0	11.4 ± 21.0
Drinking status					
No	3704 (21.0%)	861 (16.9%)	1089 (21.1%)	922 (23.3%)	832 (24.3%)
Yes	13923 (79.0%)	4219 (83.1%)	4074 (78.9%)	3042 (76.7%)	2588 (75.7%)
Aspirin using regularly					
No	9418 (53.4%)	2709 (53.3%)	2723 (52.7%)	2155 (54.4%)	1831 (53.5%)
Yes	8209 (46.6%)	2371 (46.7%)	2440 (47.3%)	1809 (45.6%)	1589 (46.5%)
Family history of colorectal cancer					
No	15655 (88.8%)	4477 (88.1%)	4562 (88.4%)	3535 (89.2%)	3081 (90.1%)
Yes	1521 (8.6%)	459 (9.0%)	457 (8.9%)	339 (8.6%)	266 (7.8%)
Possibly	451 (2.6%)	144 (2.8%)	144 (2.8%)	90 (2.3%)	73 (2.1%)
History of diabetes					
No	16563 (94.0%)	4905 (96.6%)	4883 (94.6%)	3697 (93.3%)	3078 (90.0%)
Yes	1064 (6.0%)	175 (3.4%)	280 (5.4%)	267 (6.7%)	342 (10.0%)
History of hypertension					
No	12236 (69.4%)	3547 (69.8%)	3574 (69.2%)	2746 (69.3%)	2369 (69.3%)
Yes	5391 (30.6%)	1533 (30.2%)	1589 (30.8%)	1218 (30.7%)	1051 (30.7%)
history of diverticulitis or diverticulosis					
No	16802 (95.3%)	4849 (95.5%)	4935 (95.6%)	3775 (95.2%)	3243 (94.8%)
Yes	825 (4.7%)	231 (4.5%)	228 (4.4%)	189 (4.8%)	177 (5.2%)
history of colonoscopy in past 3 years					
No	9963 (56.5%)	3120 (61.4%)	2985 (57.8%)	2102 (53.0%)	1756 (51.3%)
Yes	7664 (43.5%)	1960 (38.6%)	2178 (42.2%)	1862 (47.0%)	1664 (48.7%)
Total energy intake (kcal/d)	2087.9 ± 798.6	2251.9 ± 843.9	2077.9 ± 800.8	1984.7 ± 759.9	1979.1 ± 726.7
Animal protein (g/d)	51.7 ± 25.4	54.3 ± 23.6	53.8 ± 30.1	49.3 ± 23.9	47.2 ± 20.6
Plant protein (g/d)	30.6 ± 12.7	26.5 ± 10.0	28.7 ± 11.2	32.1 ± 12.8	37.7 ± 14.7
Total carbohydrate (g/d)	280.7 ± 104.7	287.0 ± 107.6	278.3 ± 105.8	275.8 ± 102.2	280.6 ± 101.2
Total fat (g/d)	68.3 ± 33.2	77.2 ± 35.7	68.3 ± 33.7	63.3 ± 30.7	60.8 ± 28.0
Total fiber (g/d)	24.1 ± 10.0	20.4 ± 7.8	22.8 ± 8.8	25.8 ± 10.3	29.4 ± 11.5
Red and processed meat (g/d)	78.0 ± 64.9	90.5 ± 63.5	84.1 ± 77.5	70.0 ± 56.2	59.7 ± 47.7
Fish (g/d)	29.1 ± 31.2	24.4 ± 22.4	27.8 ± 30.1	30.1 ± 32.5	36.6 ± 39.7
Poultry (g/d)	54.7 ± 48.4	47.1 ± 36.6	54.4 ± 51.0	56.3 ± 50.6	64.7 ± 54.5
Eggs (g/d)	13.2 ± 17.4	16.0 ± 19.5	13.6 ± 18.3	11.4 ± 14.4	10.3 ± 15.0
Dairy (g/d)	82.7 ± 78.4	95.6 ± 84.5	81.3 ± 76.4	76.0 ± 73.7	73.7 ± 74.4
Bean and pea (g/d)	44.3 ± 37.8	31.5 ± 22.6	38.6 ± 28.7	47.6 ± 36.0	67.9 ± 54.6
Nuts (serving/d)	0.2 ± 0.4	0.2 ± 0.2	0.2 ± 0.3	0.3 ± 0.4	0.3 ± 0.4

Values are means (standard deviation) for continuous variables and percentages for categorical variables.

### Protein diet score and colorectal adenoma

**Figure 3:**
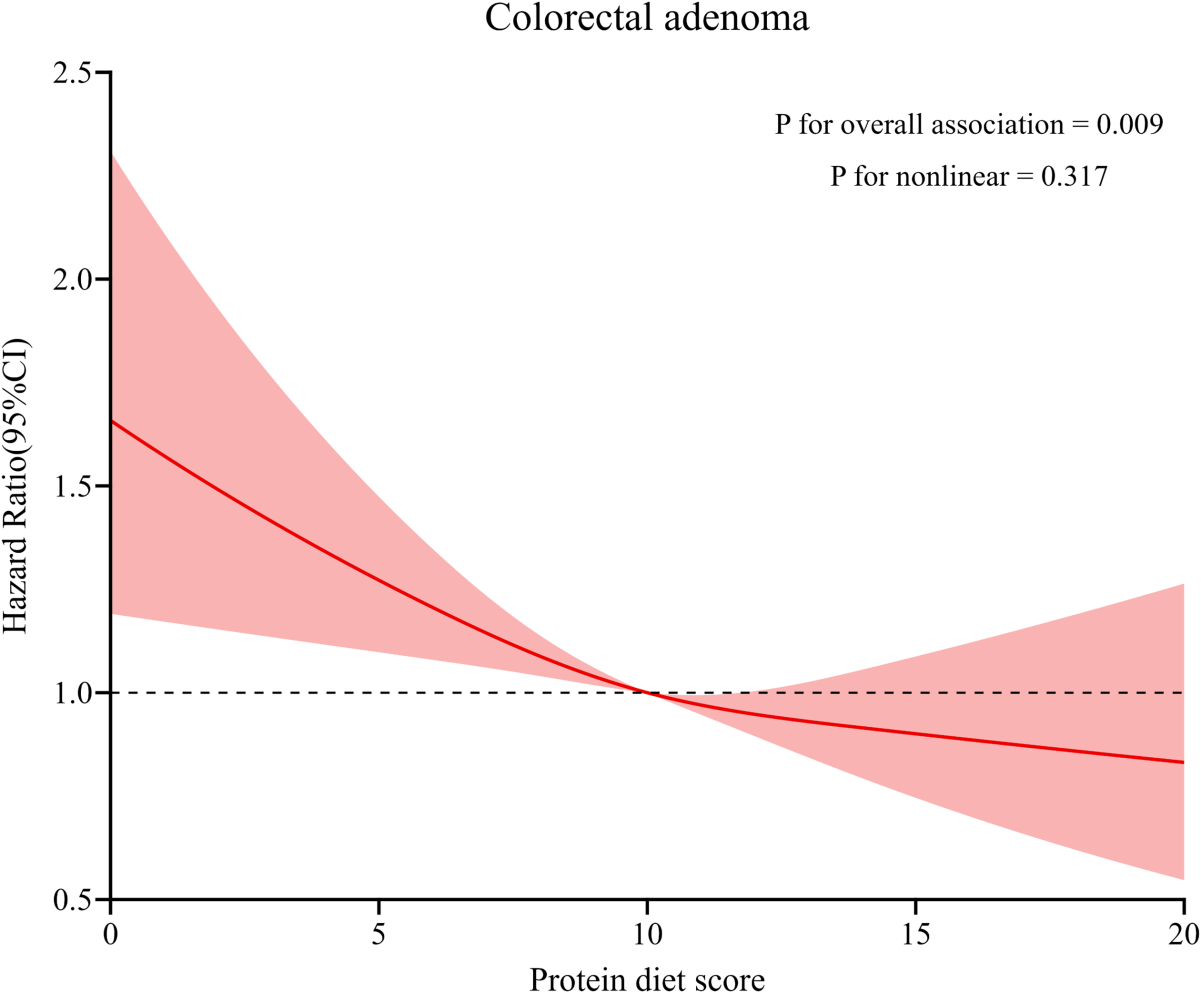
Dose–response analysis on the association of Protein Diet Score with the risk of colorectal adenoma. Hazard ratios was adjusted for age, sex, race, education levels, occupation, marital status, BMI, smoking status, pack-years smoked, drinking status, aspirin using regularly, family history of colorectal cancer, history of diabetes, history of hypertension, history of diverticulitis or diverticulosis, and history of colonoscopy in past 3 years.

During the mean follow-up period of 11.0 years, 992 newly diagnosed colorectal adenomas were identified. **Table 2** presents the HRs of the association between Protein Diet Score and risk of colorectal adenoma. In the unadjusted model, participants in the highest quartile demonstrated a lower incidence of colorectal adenoma compared with those in the lowest quartile (HR _Quartile 4 vs. Quartile 1_: 0.68; 95% CI: 0.56-0.81; P_trend_ <0.001). After comprehensive adjustment for potential confounders, the inverse association remained statistically significant with an HR of 0.81 (95% CI: 0.67-0.99; P_trend_ =0.005) comparing the highest versus lowest quartile. Restricted cubic spline analysis revealed a linear inverse relationship between Protein Diet Score and colorectal adenoma risk (P for nonlinearity =0.317; **Figure 3**). Furthermore, the PAR showed a 21% lower risk of colorectal adenoma in the highest versus lowest quartile (HR _Quartile 4 vs. Quartile 1_: 0.79; 95% CI: 0.64-0.97; P_trend_ =0.025). However, no significant association was observed between E% and colorectal adenoma risk.

**Table 2:** Hazard ratios of the association between colorectal adenoma risk and Protein Diet Score as well as its components, including E% and PAR.

Classification	Number of cases	Person-years	Incidence rate per 100 person-years (95% confidence interval)	Hazard ratio (95% confidence interval)		
				Unadjusted	Model 1^a^	Model 2^b^
Quartiles of Protein Diet Score						
Quartile 1	357	54635.3	0.65 (0.59, 0.72)	1.000 (reference)	1.000 (reference)	1.000 (reference)
Quartile 2	293	56962.7	0.51 (0.46, 0.58)	0.79 (0.68, 0.93)	0.84 (0.72, 0.98)	0.87 (0.74, 1.01)
Quartile 3	175	44431.1	0.39 (0.34, 0.46)	0.62 (0.51, 0.74)	0.67 (0.56, 0.81)	0.70 (0.59, 0.85)
Quartile 4	167	38656.8	0.43 (0.37, 0.50)	0.68 (0.56, 0.81)	0.76 (0.63, 0.92)	0.81 (0.67, 0.99)
P_trend_				<0.001	<0.001	0.005
Quartiles of E%						
Quartile 1	295	53139.8	0.56 (0.50, 0.62)	1.000 (reference)	1.000 (reference)	1.000 (reference)
Quartile 2	252	52943.1	0.48 (0.42, 0.54)	0.86 (0.73, 1.01)	0.87 (0.73, 1.02)	0.88 (0.74, 1.04)
Quartile 3	278	53288.3	0.52 (0.46, 0.59)	0.94 (0.80, 1.11)	0.95 (0.81, 1.12)	0.94 (0.80, 1.11)
Quartile 4	167	35314.7	0.47 (0.41, 0.55)	0.85 (0.70, 1.02)	0.88 (0.72, 1.06)	0.84 (0.69, 1.02)
P_trend_				0.175	0.305	0.156
Quartiles of PAR						
Quartile 1	317	51763.4	0.61 (0.55, 0.68)	1.000 (reference)	1.000 (reference)	1.000 (reference)
Quartile 2	280	52823.3	0.53 (0.47, 0.60)	0.88 (0.75, 1.03)	0.91 (0.78, 1.07)	0.96 (0.81, 1.12)
Quartile 3	255	53648.9	0.48 (0.42, 0.54)	0.79 (0.67, 0.93)	0.84 (0.71, 1.00)	0.90 (0.76, 1.07)
Quartile 4	140	36450.3	0.38 (0.33, 0.45)	0.64 (0.53, 0.79)	0.70 (0.57, 0.86)	0.79 (0.64, 0.97)
P_trend_				<0.001	<0.001	0.025

a: Model 1 was controlled with age (continuous), sex (male, female), race (white, non-white), education levels (college below, college graduate, postgraduate), occupation (not working, working, retired, other, unknown) and marital status (no, yes).

b: Model2 was additionally controlled with BMI (continuous), smoking status (never, current, former), pack-years smoked (continuous), drinking status (no, yes), aspirin using regularly (no, yes), family history of colorectal cancer (no, yes), history of diabetes (no, yes), history of hypertension (no, yes), history of diverticulitis or diverticulosis (no, yes), and history of colonoscopy in past 3 years (no, yes).

E%: protein intake as a percentage of total energy; PAR: plant-to-animal protein ratio.

### Subgroup and sensitivity analyses

In subgroup analyses (**Table 3**), we observed a more pronounced inverse association between Protein Diet Score and colorectal adenoma among participants with a history of hypertension (HR _Quartile 4 vs. Quartile 1_: 0.60; 95% CI: 0.43-0.85; P_interaction_ =0.017). The association was not modified by other pre-defined potential effect modifiers (all P_interaction_ > 0.05).

**Table 3:** Subgroup analyses on the associations of Protein Diet Score and the risk of colorectal adenoma.

Subgroup variable	Number of participates	Number of cases	HR _Quartile 4_ vs. _Quartile 1_ (95% CI)	P _interaction_
Age				0.121
≤65 years old	12820	736	0.84 (0.68, 1.05)	
>65 years old	4807	256	0.72 (0.49, 1.06)	
Sex				0.563
Male	9868	655	0.80 (0.63, 1.02)	
Female	7759	337	0.88 (0.64, 1.21)	
Smoking status				0.614
No	9379	429	0.92 (0.69, 1.22)	
Yes	8248	563	0.71 (0.54, 0.92)	
Family history of colorectal cancer				0.710
No	15655	875	0.83 (0.68, 1.02)	
Yes/Possibly	1972	117	0.65 (0.35, 1.21)	
Body mass index (kg/m2)				0.367
≤30	13888	739	0.76 (0.61, 0.95)	
>30	3739	253	0.95 (0.64, 1.40)	
Aspirin using regularly				0.132
No	9418	517	0.93 (0.72, 1.20)	
Yes	8209	475	0.70 (0.53, 0.93)	
History of hypertension				0.017
No	12236	676	0.95 (0.75, 1.19)	
Yes	5391	316	0.60 (0.43, 0.85)	

Fully adjusted model was controlled with age (continuous), sex (male, female), race (white, non-white), education levels (college below, college graduate, postgraduate), occupation (not working, working, retired, other, unknown), marital status (no, yes), BMI (continuous), smoking status (never, current, former), pack-years smoked (continuous), drinking status (no, yes), aspirin using regularly (no, yes), family history of colorectal cancer (no, yes), history of diabetes (no, yes), history of hypertension (no, yes), history of diverticulitis or diverticulosis (no, yes), and history of colonoscopy in past 3 years (no, yes).

Sensitivity analyses demonstrated the robustness of our findings (**Table 4**). The inverse associations remained significant after sequential exclusion of participants with a history of diverticulitis (HR: 0.83; 95% CI: 0.68-1.00), history of diabetes (HR: 0.77; 95% CI: 0.63-0.95), family history of colorectal cancer (HR: 0.83; 95% CI: 0.68-1.02), and cases occurring within the first two (HR: 0.81; 95% CI: 0.67-0.99) and four years (HR: 0.78; 95% CI: 0.63-0.97) of follow-up, with all P_trend_ < 0.05. Furthermore, the inverse association persisted after additional adjustment for carbohydrate and fat intake in Model 2 (HR _Quartile 4 vs. Quartile 1_: 0.78; 95% CI: 0.62-0.98; P_trend_ =0.009). When Model 2 was further adjusted for protein-rich foods including red and processed meat, fish, poultry, dairy products, eggs, soy and soy products, peas, nuts and seeds, and cereals, the association remained similar (HR _Quartile 4 vs. Quartile 1_: 0.83; 95% CI: 0.67-1.03; P_trend_ =0.026).

**Table 4:** Sensitivity analyses on the between Protein Diet Score and the risk of colorectal adenoma^a^.

Categories	HR _Quartile 4 vs. Quartile 1_ (95% CI) ^a^	P_trend_
Primary analysis	0.81 (0.67, 0.99)	0.005
Excluded participants with history of diverticulitis or diverticulosis	0.83 (0.68, 1.00)	0.009
Excluded participants with a history of diabetes	0.77 (0.63, 0.95)	0.002
Excluded participants with family history of colorectal cancer	0.83 (0.68, 1.02)	0.015
Additionally adjusted for carbohydrate, and fat intake in model 2	0.78 (0.62, 0.98)	0.009
Additionally adjusted for foods containing protein nutrients in model 2^b^	0.83 (0.67, 1.03)	0.026
Excluded cases observed within the first 2 years of follow-up	0.81 (0.67, 0.99)	0.005
Excluded cases observed within the first 4 years of follow-up	0.78 (0.63, 0.97)	0.002

a: Fully adjusted model was controlled with age (continuous), sex (male, female), race (white, non-white), education levels (college below, college graduate, postgraduate), occupation (not working, working, retired, other, unknown), marital status (no, yes), BMI (continuous), smoking status (never, current, former), pack-years smoked (continuous), drinking status (no, yes), aspirin using regularly (no, yes), family history of colorectal cancer (no, yes), history of diabetes (no, yes), history of hypertension (no, yes), history of diverticulitis or diverticulosis (no, yes), and history of colonoscopy in past 3 years (no, yes).

b: further adjusted for protein-rich foods including red and processed meat, fish, poultry, dairy products, eggs, soy and soy products, peas, nuts and seeds, and cereals.

## Discussion

In this prospective study, we identified an inverse association between higher Protein Diet Score and the incidence of colorectal adenoma in middle-aged and elderly populations. Further analysis of the Protein Diet Score components revealed that the PAR was negatively correlated with colorectal adenoma incidence, while E% showed no significant association. In subgroup analyses, we found that hypertension history demonstrated significant interaction with the relationship between Protein Diet Score and colorectal adenoma incidence. A series of sensitivity analyses strengthened the robustness of our findings.

Previous epidemiological studies on dietary protein and disease primarily focused on total protein intake or protein from specific sources, lacking comprehensive assessment of both protein intake and sources. For instance, a prospective analysis using the UK Biobank found inverse associations between protein intake from dairy products and milk with colorectal cancer risk (17). A meta-analysis demonstrated that higher total protein intake was associated with lower all-cause mortality risk, and substituting plant for animal protein sources might be linked to longevity (18). In contrast, the Protein Diet Score, a comprehensive system incorporating protein intake and sources, enables thorough evaluation of dietary protein intake and origins. The relationship between dietary protein and colorectal adenoma risk remains inconclusive. A cross-sectional study in Korea examining the association between protein intake and colorectal adenoma risk found no significant relationship after comprehensive adjustment for risk factors (7). To clarify this relationship and investigate the impact of a more comprehensive protein assessment approach on colorectal adenoma, we conducted this study analyzing the association between Protein Diet Score and colorectal adenoma risk.

The impact of dietary protein on intestinal homeostasis is complex, encompassing both potential protective effects and risks. Indeed, chronic inflammation is recognized as a crucial pathway in cancer development, and research indicates that dietary protein may influence this process by modulating colorectal inflammation and immune responses. Studies have shown that dietary protein may promote the accumulation and clonal selection of CD4+ T cells within the intestinal epithelium. This action helps maintain epithelial-adaptive CD4+ T cell populations under intestinal homeostasis, which may be critical for preventing excessive immune responses and related diseases (8). Additionally, amino acids, the fundamental building blocks of proteins, may influence intestinal health by modulating key cellular signaling pathways. Specifically, certain amino acids may reduce inflammation by inhibiting the NF-κB signaling pathway while alleviating oxidative stress through activation of the Nuclear factor erythroid 2-related factor 2 (Nrf2) signaling pathway (9, 10). While protein can mitigate intestinal immune inflammation, excessive intake may increase health risks, primarily by elevating IGF-I levels. IGF-I plays a crucial role in cancer development; upon binding to its receptor, it activates signaling pathways including PI3K/Akt and MAPK, promoting excessive colorectal cell proliferation and thereby increasing the risk of intestinal tumors (11, 12). Therefore, moderate dietary protein intake is essential for maintaining intestinal homeostasis and long-term intestinal health. These findings emphasize that balanced protein consumption can effectively support optimal intestinal function.

The source of dietary protein is crucial for its intestinal metabolism and health effects. Animal experiments and *in vitro* studies have shown that dietary proteins are fermented by gut microbiota in the gastrointestinal tract, producing various compounds including ammonia, phenols, amines, hydrogen sulfide, and short-chain fatty acids (SCFAs) (19). These metabolites may exert toxic effects, including damaged colonic epithelial cell structure and metabolic function, significantly thinned mucosal barrier, and increased colonic permeability (20–24). Animal-sourced proteins demonstrate higher digestibility compared to plant-sourced proteins (25). Given that protein malabsorption may lead to harmful substance production in the intestine, we need to reassess protein intake strategies. This may help balance protein digestion and absorption with gut microbial metabolic activity.

Protein, as an essential macronutrient for humans, is primarily obtained from various dietary sources. Given this fact, most research tends to investigate the holistic effects of protein-containing foods rather than examining protein’s impact on human health in isolation. For example, while red meat and processed meat products are significant protein sources, studies have confirmed their potential role in increasing gastrointestinal cancer risk (26–28). These adverse effects may be primarily attributed to nitrites, nitrosamines, and compounds formed during high-temperature cooking such as polycyclic aromatic hydrocarbons (PAHs) and heterocyclic amines (HCAs), rather than the protein itself (29, 30).

Our study demonstrated that the association between Protein Diet Score and colorectal adenoma risk was more pronounced in specific populations. Notably, individuals with a history of hypertension exhibited a stronger inverse association between Protein Diet Score and colorectal adenoma risk. This enhanced association may be attributed to multiple interacting factors. Initially, hypertensive patients are typically advised to adhere to specific dietary patterns, such as the Dietary Approaches to Stop Hypertension (DASH) diet, which promotes the consumption of fruits, vegetables, whole grains, and lean proteins (31). This combination of multiple nutrients may synergistically maintain colorectal homeostasis, thereby reducing adenoma risk (32). Hypertensive patients typically place greater emphasis on comprehensive lifestyle management. Beyond dietary control, they tend to increase physical activity and maintain weight control. These integrated health behaviors may work synergistically to enhance the protective effects of dietary protein against colorectal adenoma risk (33, 34).

Our study offers several strengths. First, it provides novel evidence of the relationship between Protein Diet Score and colorectal adenoma risk, addressing previous knowledge gaps and offering new insights into protein’s impact on intestinal health. Additionally, the Protein Diet Score comprehensively assesses both protein intake and quality, helping guide individuals in optimizing their dietary protein quantity and composition. Second, our prospective cohort design with a large population sample enhances the reliability and generalizability of our findings. Third, to minimize potential bias, we conducted thorough adjustments for a wide range of confounding factors in our statistical analyses. Furthermore, we conducted a detailed analysis of the associations between the two components of the Protein Diet Score and colorectal adenoma. Results showed that only PAR exhibited a significant inverse association with colorectal adenoma, further underscoring the importance of evaluating both the source and quantity of dietary protein comprehensively. Fourth, a series of sensitivity analyses confirmed the robustness of our results.

However, our study has several limitations. First, approximately 14,000 participants in the PLCO trial did not complete valid DQXs. This substantial non-response may not accurately reflect the distribution of dietary exposures. Second, although the DQX has been validated as an effective dietary questionnaire, dietary history information was self-reported, potentially introducing non-differential bias. Third, despite comprehensive control of potential confounders based on previous literature and clinical knowledge, we cannot completely rule out residual confounding from unmeasured factors.

## Conclusion

Our findings indicate that higher Protein Diet Score is associated with reduced colorectal adenoma incidence among middle-aged and elderly Americans, with similar findings observed for the PAR. These results provide important evidence for optimizing protein intake and source composition to promote intestinal health. However, given that this study primarily focused on the US population, future research should investigate the association between protein intake patterns and colorectal adenoma risk in diverse populations with varying dietary habits, genetic backgrounds, and environmental factors.

## Data Availability

The datasets presented in this study can be found in online repositories. The names of the repository/repositories and accession number(s) can be found in the article/**Supplementary Material**.
