# Correction: Association between protein diet score and colorectal adenomas risk: a prospective study

**DOI:** 10.3389/fimmu.2025.1690311

**Published:** 2025-09-05

**Authors:** Yangpiaoyi Shi, Zhiquan Xu, Wanhao Tan, Hang Liu, Qi Wei, Yaxu Wang, Ling Xiang, Linglong Peng, Haitao Gu

**Affiliations:** ^1^ Department of Gastrointestinal Surgery, The Second Affiliated Hospital of Chongqing Medical University, Chongqing, China; ^2^ Department of Hepatobiliary Surgery, The First Affiliated Hospital of Chongqing Medical University, Chongqing, China; ^3^ Department of Clinical Nutrition, The Second Affiliated Hospital of Chongqing Medical University, Chongqing, China

**Keywords:** protein, protein diet score, colorectal adenoma, epidemiology, prospective study

Affiliation 1 “Department of Gastrointestinal Surgery, The Second Affiliated Hospital of Chongqing Medical University, Chongqing, China” was omitted for author Zhiquan Xu. This affiliation has now been added for author Zhiquan Xu.

The original version of this article has been updated.

