# Supplementary material for: Association between protein diet score and colorectal adenomas risk: a prospective study
Source: Front Immunol. 2025 Jun 26;16:1529011. doi: 10.3389/fimmu.2025.1529011 (PMC12240751; doi:10.3389/fimmu.2025.1529011)
Supplement: Supplementary file 1 [file Table1.docx]

**Association Between Protein Diet Score And Colorectal Adenomas Risk: A Prospective Study**

Yangpiaoyi Shi ^1, #^, Zhiquan Xu ^2, #^, Wanhao Tan ^1^, Hang Liu ^1^, Qi Wei ^1^, Yaxu Wang ^1^, Ling Xiang ^3^, Linglong Peng ^1^, Haitao Gu ^1,^ *

1 *Department of* *Gastrointestinal Surgery, The Second Affiliated Hospital of Chongqing Medical University, Chongqing, China.*

2 *Department of Hepatobiliary Surgery, the First Affiliated Hospital of Chongqing Medical University, Chongqing, China*

3 *Department of Clinical Nutrition, The* *Second Affiliated Hospital of Chongqing Medical University, Chongqing, China.*

Correspondence authors:

Haitao Gu, Department of Gastrointestinal Surgery, The Second Affiliated Hospital of Chongqing Medical University, No.288 Tianwen Avenue, Nan'an District, Chongqing, 400010, China. fax: +86 023 6288 7521. E-mail addresses: [ght302211@cqmu.edu.cn](mailto:306359@hospital.edu.cn)

^#^ These authors contributed equally to this work.

**Table S1**. Distribution of covariates with missing data before and after imputation^1^

| **Variable** | **Before imputation** | **After imputation** ^a^ | **Number (%) with missing data** |
| --- | --- | --- | --- |
| **Race** |  |  | 0.02% |
| White | 15952 (90.5%) | 15955 (90.5%) |  |
| Non-White | 1672 (9.5%) | 1672 (9.5%) |  |
| **Marital status** |  |  | 0.08% |
| Married | 14343 (81.4%) | 59566 (79.8%) |  |
| Unmarried | 3270 (18.6%) | 15103 (20.2%) |  |
| **Education** |  |  | 0.10% |
| College below | 10678 (60.6%) | 10696 (60.7%) |  |
| College graduate | 3263 (18.5%) | 3263 (18.5%) |  |
| Postgraduate | 3668 (20.8%) | 3668 (20.8%) |  |
| **Body mass index at baseline (kg/m2)** | 27.1±4.6 | 27.1±4.6 | 0.93% |
| **Smoking status** |  |  | 0.02% |
| No | 9375 (53.2%) | 9379 (53.2%) |  |
| Current | 970 (5.5%) | 970 (5.5%) |  |
| Former | 7278 (41.3%) | 7278 (41.3%) |  |
| **Pack-years smoked** | 14.1±23.6 | 13.9±23.5 | 0.89% |
| **Aspirin using regularly** |  |  | 0.31% |
| No | 9364 (53.3%) | 9418 (53.4%) |  |
| Yes | 8209 (46.7%) | 8209 (46.6%) |  |
| **Family history of colorectal cancer** |  |  | 0.64% |
| No | 15542 (88.7%) | 15655 (88.8%) |  |
| Yes | 1521 (8.7%) | 1521 (8.6%) |  |
| Possibly | 451 (2.6%) | 451 (2.6%) |  |
| **History of diabetes** |  |  | 0.02% |
| No | 16560 (94.0%) | 16563 (94.0%) |  |
| Yes | 1064 (6.0%) | 1064 (6.0%) |  |
| **History of hypertension** |  |  | 0.05% |
| No | 12228 (69.4%) | 12236 (69.4%) |  |
| Yes | 5391 (30.6%) | 5391 (30.6%) |  |
| **history of diverticulitis or**  **diverticulosis** |  |  | 0.09% |
| No | 16786 (95.3%) | 16802 (95.3%) |  |
| Yes | 825 (4.7%) | 825 (4.7%) |  |
| **history of colonoscopy in past 3 years** |  |  | 3.35% |
| No | 9372 (55.0%) | 9963 (56.5%) |  |
| Yes | 7664 (45.0%) | 7664 (43.5%) |  |

^1^Values are mean (standard deviation) or counts (percentage) as indicated.
